# Accuracy and safety of a new robotic arm for both femoral and acetabular side in total hip arthroplasty: a cadaveric study

**DOI:** 10.1186/s13018-023-04263-w

**Published:** 2023-11-03

**Authors:** Xinzhe Lu, Zian Zhang, Wenzhe Wang, Hao Xu, Haining Zhang

**Affiliations:** Department of Joint Surgery, The Affiliated Hospital of Qingdao University, Qingdao, 266100 Shandong China

**Keywords:** Total hip arthroplasty (THA), Robot, Cadaveric study

## Abstract

**Background:**

To investigate the accuracy and safety of a newly constructed robotic arm which can cover the whole process of THA, we performed a series of robot-assisted total hip replacement on the cadaver.

**Methods:**

Fifteen frozen cadaveric specimens (30 hips) were used for this study. In this investigation, united hip system and Longwell robotic-assisted system were used. The entire lower limb was CT scanned prior to surgery. The 3D model was produced based on CT data; the site of the prosthesis, including acetabular anteversion, inclination angle, and the position of femoral prosthesis, was planned. With the assistance of a robotic arm, the surgeon changed the parameters based on the preoperative plan and the actual condition during surgery, and completed the whole procedure. Following surgery, we measured the acetabular anteversion angle, acetabular inclination angle, femur anteversion angle, combined anteversion angle, stem angulation, and canal fill ratio.

**Results:**

The parameters proved that the acetabular anteversion angle was 16.85 ± 3.00°, the acetabular inclination angle was 40.38 ± 5.37°, femur anteversion angle was 15.90 ± 9.01°, combined anteversion angle was 32.75 ± 9.03°, stem angulation was 1.84 ± 0.99°, and leg length discrepancy was 2.47 ± 1.43 mm. The canal fill ratio (CFR) of femoral prosthesis of osteotomy line in sagittal section is 99.72 ± 1.54% and in coronal section is 62.94 ± 8.91%; below osteotomy line 2.5 cm in sagittal section is 100.00% and in coronal section is 81.48 ± 12.94%; below osteotomy line 7.5 cm in sagittal section is 59.51 ± 12.94% and in coronal section is 89.79 ± 11.13%; femoral shaft isthmus in sagittal section is 56.41 ± 13.80% and in coronal section is 84.95 ± 15.17%.

**Conclusion:**

The accuracy and safety of this novel robotic arm are suitable for preparing both the acetabular and femoral sides, providing evidence for clinical trial.

## Introduction

Total hip arthroplasty (THA) is an effective treatment for end-stage hip diseases such as avascular necrosis of the femoral head and congenital dysplasia of the hip, relieving pain and restoring hip function [1]. It is anticipated that the number of THA in the USA would increase by 71% to 635,000 by the year 2030 [2].

Although total hip arthroplasty is currently well developed, and the postoperative patient satisfaction rate is just 89% [3]. The main cause of postoperative dissatisfaction is postoperative pain [4], such as impact pain caused by activity. Malposition of prosthesis and dislocation following surgery are also among the leading causes of revision [5–7].

**Fig. 1:**
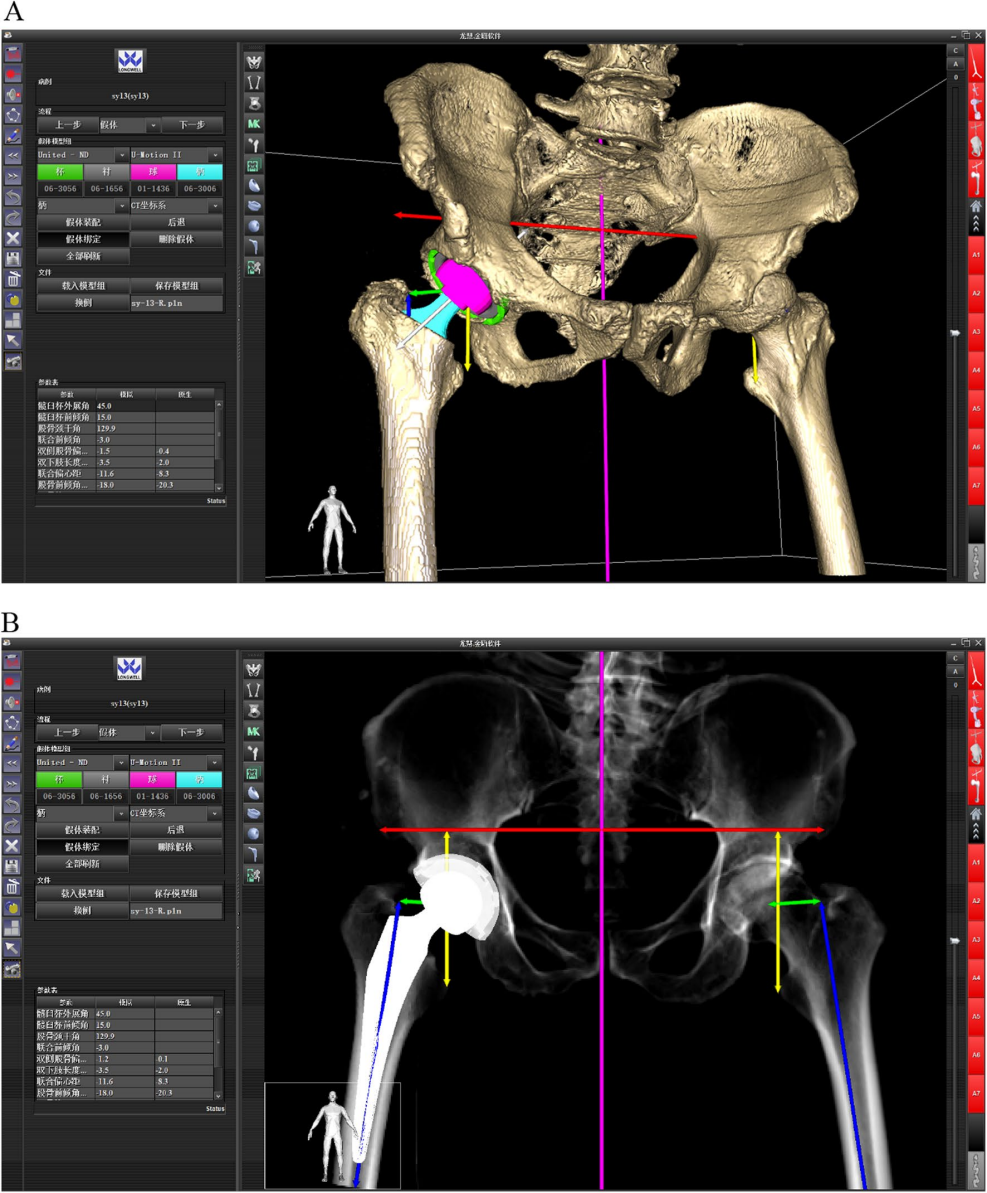
The operation interface **A** and the X-ray film simulated according to the intraoperative data **B**

During conventional total hip arthroplasty, surgeon’s experience with or without manual instruments, which is irreplaceable and has a long learning curve [8]. Computer-assisted techniques like navigation or robot-assisted THA have proven to be safe and reliable methods [9, 10], and can enhance the accuracy of the operation [11, 12]. Robot-assisted THA can better restore lower limb length and enhance hip-specific function [9, 12–14]. Commercial robots can currently only operate on the acetabular side. In addition to producing the acetabulum like previous surgical robots on the market, this new type of robot can also produce the femoral side without shifting the location of the surgeon and the robot. In addition, a simulated X-ray film is produced based on the data obtained during surgery displaying the current placement of the prosthesis (Fig. 1). This study’s primary goal is to confirm the accuracy and safety of this robot-assisted system.

## Materials and methods

Ethical approval for this study (QYFYEC 2020-017-01) was provided by the Ethical Committee NAC of Affiliated Hospital of Qingdao University, Qingdao, China, on November 04, 2020.

Fifteen frozen cadaveric specimens (30 hips) were used in this study. Eight of the 15 cadavers were males. All of cadavers’ knee joint, hip joint and ankle joint are intact. Two groups of experienced teams perform total hip arthroplasty with the assistance of TRex-R system (Longwell Corp., China) robot. All operators are familiar with the operation flow and principle of Longwell robot and have experience of the operation of robotic-assisted arm. At the same time, each operator is a skilled joint surgeon, and each has at least 50 cases of total hip arthroplasty experience every year. United hip system (United Orthopedic Corp., Hsinchu, Taiwan) was used to be the implanted prosthesis in this test. The purpose of the operation was to accurately placed the acetabular and femur prosthesis within a defined range, and after placement, both the acetabular and femur prosthesis were marked and located with a probe.

### Robotic procedure

CT scan of the full length of both lower limbs was obtained and the data were then imported to the preoperative planning system (Longwell, Shanghai, China) before the operation. The experimental scheme was planned on the basis of the reconstruction model, including acetabular prosthesis size, inclination, anteversion angle and depth, femoral prosthesis size, femoral anteversion, stem angulation and depth. According to the Lewinnek safety zone, the acetabular anteversion and inclination were targeted to 15° ± 10°and 40° ± 10°, respectively. And the target range for combined anteversion was 25°–50° [15].

The cadaver was fixed in the lateral position on the test bench, install the base target, register the host, and confirm that the robot and manipulator can be recognized by NDI Polaris system. The pelvic tracker was fixed at the iliac crest, and the direct anterior lateral approach was performed to expose the hip joint. The checkpoint was set at the lateral side of the proximal femur, and then, registration on the femoral side was carried out like Mako “Enhance” procedure. Osteotomy along the femoral neck at the level according to the preoperative plan was performed by the saw attached at the robotic arm, and then, femoral head was removed. The Registration process of the acetabular bone was carried out. With the assistance of the reamer attached at the robot arm, the acetabular cartilage and subchondral bone was removed to the planned level. The power will immediately shut off automatically if the angle or depth of the acetabular file exceeds the safe range. After the mold test parameters have been determined to be suitable, the acetabular prosthesis is inserted with the aid of a robot arm, and the corresponding polyethylene lining is applied. The proximal end of the femur was exposed and the canal was opened by the box chisel attached the robotoc arm. Broaching the femoral canal, as well as reaming and preparation the proximal femur, trial insertion and prosthesis insertion, was all performed with the aid of robotic arm, by using different connection instruments connected at the end of the arm.

### Navigation and measurement

In order to verify the accuracy of the position of the prosthesis after the operation, CT scan of the total lower limbs was accomplished. The postoperative CT scan was imported into the measurement program to determine the acetabular anteversion, acetabular inclination, femoral anteversion, length of both lower extremities, and the filling rate of the femoral prosthesis. In order to keep the precision as high as possible, the measurement was carried out by two experienced orthopedic surgeons who did not participate in the experiment. If the difference between the two results was too large (≥ 5°or ≥ 5 mm), it was measured by the third orthopedic surgeon who did not participate in the experiment. The final result was selected for the data close to the average of two of the three. In order to ensure the reliability of the data, the probe was used to locate the prosthesis during the operation, and the C-arm machine was used to take a plain film of the lower limbs, which was compared with the simulated X-ray.

### Statistical analysis

All statistical analyses were performed using SPSS version 26.0 (IBM Corp., Armonk, NY). For continuous variables, the data were presented as means ± standard deviations (SD).

## Results

In this study, the operation time was 103.3 ± 24.0 min, registration time was 9.7 ± 2.9 min, acetabular registration accuracy was 1.1 ± 0.4 mm and femur registration accuracy was 1.3 ± 0.4 mm, and with the median was 104.0 min, 9.0 min, 1.0 mm and 1.9 mm (Table 1).

**Table 1:** Operation time and registration accuracy (*n* = 30)

	Mean ± SD	Median
Operation time (min)	103.33 ± 24.03	104.00
Registration time (min)	9.67 ± 2.90	9.00
Acetabular registration accuracy (mm)	1.09 ± 0.37	0.97
Femur registration accuracy (mm)	1.25 ± 0.42	1.18

The acetabular inclination angle measured by CT was 40.38 ± 5.37°, acetabular anteversion angle was 16.85 ± 3.00°, femoral anteversion angle was 15.90 ± 9.01°, combined anteversion angle was 32.75 ± 9.03°, femoral angulation was 1.84 ± 0.99° and leg length discrepancy was 2.47 ± 1.43 mm, and with the median was 40.35°, 16.35°, 16.00°, 33.10°, 2.00°, 2.55 mm (Table 2).

**Table 2:** CT data of acetabular and femoral prosthesis (*n* = 30)

	Mean ± SD	Median
Acetabular inclination angle (°)	40.38 ± 5.37	40.35
Acetabular anteversion angle (°)	16.85 ± 3.00	16.35
Femoral anteversion angle (°)	15.90 ± 9.01	16.00
Combined anteversion angle (°)	32.75 ± 9.03	33.10
Femoral angulation (°)	1.84 ± 0.99	2.00
Leg length discrepancy (mm)	2.47 ± 1.43	2.55

The canal fill ratio (CFR) of the sagittal section of the femoral prosthesis at the osteotomy line was 99.72 ± 1.54%, with a median of 100.00%. The CFR of the coronal section of the femoral prosthesis at the osteotomy line was 62.94 ± 8.91%, with a median of 63.55%. The CFR of the femoral prosthesis sagittal section 2.5 cm from the osteotomy line was 100%. The CFR of the femoral prosthesis coronal section 2.5 cm from the osteotomy line was 81.48 ± 12.94%, with a median of 84.51%. CFR of femoral prosthesis sagittal section off the osteotomy line 7.5 cm was 59.51 ± 12.94%, with a median value of 60.30%. The CFR of the coronal section of the femoral prosthesis 7.5 cm from the osteotomy line was 89.79 ± 11.13%, with a mean of 94.19%. The CFR of the sagittal section of the femoral prosthesis at the femoral isthmus was 56.41 ± 13.80%, with a median of 53.45%. The CFR of the coronal section of the femoral prosthesis at the femoral isthmus was 84.95 ± 15.17%, with a median of 86.93% (Table 3).

**Table 3:** Canal fill ratio of femoral prosthesis (%) (*n* = 30)

	Mean ± SD	Median
*Osteotomy line*		
Sagittal section	99.72 ± 1.54	100.00
Coronal section	62.94 ± 8.91	63.55
*2.5 cm off osteotomy line*		
Sagittal section	100.00	100.00
Coronal section	81.48 ± 12.94	84.51
*7.5 cm off osteotomy line*		
Sagittal section	59.51 ± 12.94	60.30
Coronal section	89.79 ± 11.13	94.19
*Isthmus of femoral*		
Sagittal section	56.41 ± 13.80	53.45
Coronal section	84.95 ± 15.17	86.93

Among the 30 specimens, the CFR of the femur in the sagittal section of the osteotomy line was > 80% in 30 cases and in 0 cases for coronal section. The CFR of femur sagittal section at 2.5 cm off the osteotomy line was > 80% in 30 cases and in 20 cases (66.67%) for coronal section. The CFR of femur sagittal section at 7.5 cm off the osteotomy line was > 80% in 1 case (3.33%) and in 21 cases (70.00%) for coronal section. The CFR of femoral isthmus in sagittal section was > 80% in 1 case (3.33%) and in 18 cases (60.00%) for coronal section (Table 4).

**Table 4:** Satisfaction of femoral prosthesis CFR (*n* = 30)

	Sagittal section > 80%	Coronal section > 80%
Osteotomy line (%)	30 (100)	0
2.5 cm off osteotomy line (%)	30 (100)	20(66.67)
7.5 cm off osteotomy line (%)	1 (3.33)	21(70.00)
Isthmus of femoral (%)	1 (3.33)	18(60.00)

*CFR* Canal fill ratio

## Discussion

**Fig. 2:**
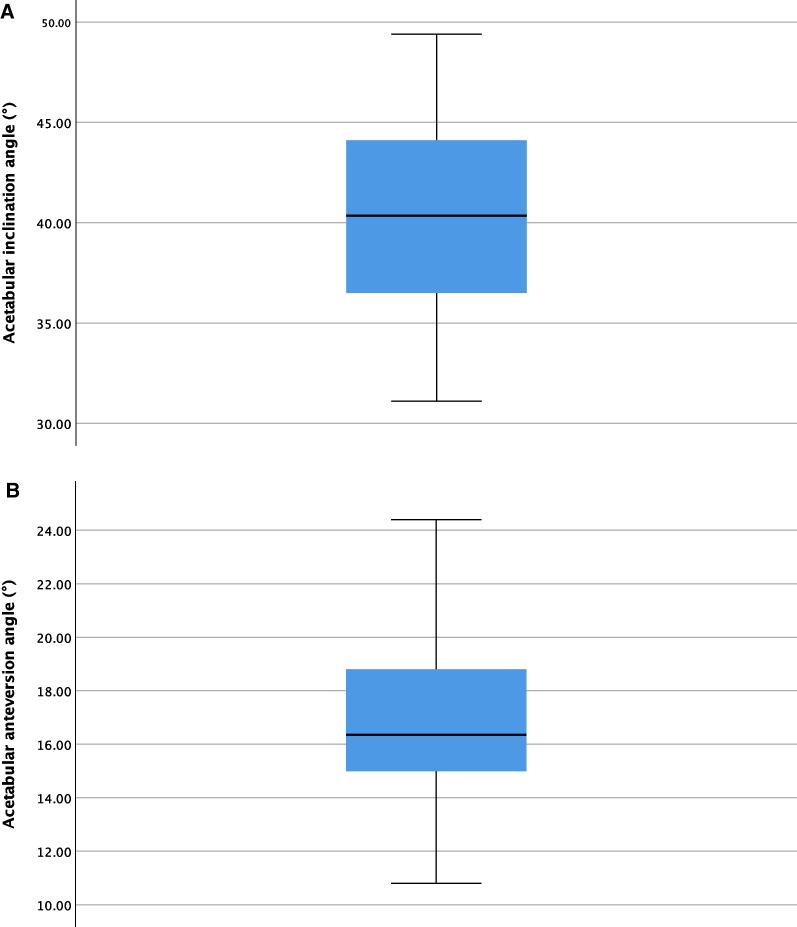
CT data of acetabular inclination angle **A** and acetabular anteversion angle **B**

The aim of this study is to verify the safety and precision of the new robot system who can cover the femoral side during THA procedure, which maybe the first robotic arm with the function for preparing the femoral canal, as well as complete the osteotomy of femoral neck. As the classic safe range for acetabular side, Lewinnek safety zone [16] points out that the anteversion of acetabular should be 15° ± 10°. The inclination angle of acetabulum should be 40° ± 10°. The plan of this trial was also planned according to this target zone. Postoperative assessment of anteversion and inclination of all acetabular sockets were found to be within these zones (Fig. 2). And there was no fracture in all the cadavers.

A study by Abdel even found that many dislocations occur within the Lewinnek safety zone [17]. With the development of new technology and the progress of ideas, more and more people advocate the use of combined anteversion to replace the Lewinnek safety zone [15, 18–20]. The factors of the femoral stalk were emphasized in this method, and with the development of navigation technology, the pelvic tilt will be measured more accurately [15, 21–23]. And our robot-assisted arm opens the door to apply a more patient-focused hip arthroplasty considering patient-specific anatomy and the interplay of both components, acetabular cup and femoral stem. With the help of robot arm, 23 of the 30 sets of data (76.67%) fall within our target range of 25°–50°.

The varus tilt of the femoral prosthesis is the most important case leading to the subsidence of the femoral prosthesis [24]. A study of Leiss found that if the varus tilt is more than 3°, it will increase the risk of femoral prosthesis sinking [25]. The stem angulation of femur assisted by robotic arm was 1.84 ± 0.99°, and the median was 2.00°, all of 30 sets of data were < 3°.

Prosthesis revision is related to prosthesis instability and aseptic loosening [26, 27], and loosening of the femoral stalk is related to poor filling rate of the femur [28]. A study of Tezuka pointed out that the filling rate of proximal femur is satisfactory when the rate is ≥ 80% [29]. Both Hwang and Streit have proved that when the filling rate of the femur is less than 80%, it may lead to aseptic loosening or sinking of the femoral stalk [30, 31]. The filling rate of all the prostheses on the median sagittal section of the osteotomy line and at the 2.5 cm off the osteotomy line were more than 80%. The filling rate was > 80% in 21 cases (70%) on the coronal section of the osteotomy line 7.5 cm, and > 80% in 18 cases (60%) on the coronal section of the isthmus of the femoral shaft (Table 4).

Periprosthetic fracture is one of the causes for early femoral revision [32]. A recent study by Alpaugh found that the risk of periprosthetic fractures increased when the femoral medullary cavity was paired with a smaller femoral prosthesis [33]. Alpaugh’s study linked the canal fill and the femoral angulation to periprosthetic fractures.

A study of 51,345 revision by Bozic et al. found that 21,047 cases (41.1%) had all-component revision and 6,738 cases (13.2%) had femoral component revision [34], accounting for 54.3% of THA revision; it means that more than half of the revision operations involved femoral prosthesis. However, a study by Brown showed that revision surgery of femoral prosthesis is often complicated due to the poor bone stock or the difficulty to remove prosthesis, which affecting the effect of surgery [35].

It can be seen that the placement and size of femoral prosthesis also have an important impact on the success of THA surgery. However, at present, the auxiliary navigation system on the market can only assist the placement of the acetabular prosthesis, and it still needs to be handled manually on the femoral side.

**Fig. 3:**
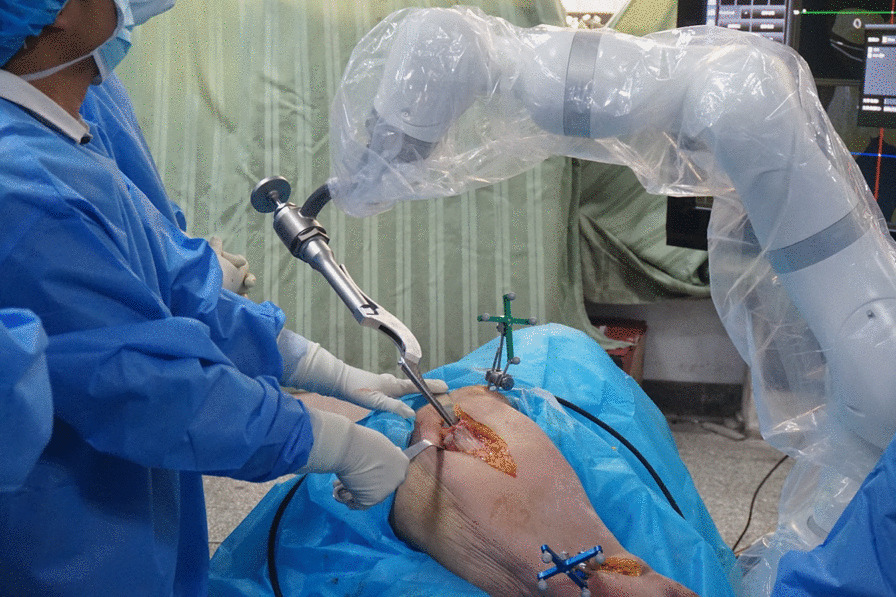
Robotic arm-assisted femoral side in surgery

The robot is a 7-axis manipulator, which breaks through the technical difficulties, so that the operation of the acetabular and femoral side can be completed without changing the position of the machine, and the size, angle and depth of the prosthesis can be monitored in real time (Fig. 3). And there is no need to repeat the process of rough registration and fine registration, which means that all surgical operations can be done in one registration.

The new designed robot arm was safe and accurate for both acetabular and femoral sides during total hip arthroplasty. As the first robotic-assisted system that can complete the operation on femoral side, this new robotic system is a meaningful attempt. Of course, this experiment also has some limitations, such as unable to count the amount of blood loss, the balance effect on soft tissue is not obvious, and unable to obtain follow-up data under the postoperative weight-bearing state. However, this trial provides a guarantee of safety and reliability for the following clinical trials, and the limitations of the trial will be resolved at the clinical environment.

## Data Availability

All data generated or analyzed during this study are included in this published article.

## References

[1] Higgins BT, Barlow DR, Heagerty NE, Lin TJ (2015). Anterior versus posterior approach for total hip arthroplasty, a systematic review and meta-analysis. J Arthroplast.

[2] Sloan M, Premkumar A, Sheth NP (2018). Projected volume of primary total joint arthroplasty in the U.S., 2014 to 2030. J Bone Jt Surg Am.

[3] Halawi MJ, Jongbloed W, Baron S, Savoy L, Williams VJ, Cote MP (2019). Patient dissatisfaction after primary total joint arthroplasty: the patient perspective. J Arthroplast.

[4] Anakwe RE, Jenkins PJ, Moran M (2011). Predicting dissatisfaction after total hip arthroplasty: a study of 850 patients. J Arthroplast.

[5] Gwam CU, Mistry JB, Mohamed NS, Thomas M, Bigart KC, Mont MA (2017). Current epidemiology of revision total hip arthroplasty in the united states: national inpatient sample 2009 to 2013. J Arthroplast.

[6] Katz JN, Wright EA, Wright J, Malchau H, Mahomed NN, Stedman M (2012). Twelve-year risk of revision after primary total hip replacement in the U.S. medicare population. J Bone Jt Surg Am.

[7] Rowan FE, Benjamin B, Pietrak JR, Haddad FS (2018). Prevention of dislocation after total hip arthroplasty. J Arthroplast.

[8] Burnham RR, Kiernan H, Ortega LF, Wesolowski M, Tauchen A, Russo M (2022). Defining the learning curve of anterior total hip arthroplasty after fellowship-specific training. J Am Acad Orthop Surg.

[9] Perets I, Walsh JP, Close MR, Mu BH, Yuen LC, Domb BG (2018). Robot-assisted total hip arthroplasty: clinical outcomes and complication rate. Int J Med Robot.

[10] Tanino H, Mitsutake R, Takagi K, Ito H (2023). Does a commercially available augmented reality-based portable hip navigation system improve cup positioning during THA compared with the conventional technique? A randomized controlled study. Clin Orthop Relat Res.

[11] Banerjee S, Cherian JJ, Elmallah RK, Pierce TP, Jauregui JJ, Mont MA (2016). Robot-assisted total hip arthroplasty. Expert Rev Med Dev.

[12] Clement ND, Gaston P, Bell A, Simpson P, Macpherson G, Hamilton DF (2021). Robotic arm-assisted versus manual total hip arthroplasty. Bone Jt Res.

[13] Ando W, Takao M, Hamada H, Uemura K, Sugano N (2021). Comparison of the accuracy of the cup position and orientation in total hip arthroplasty for osteoarthritis secondary to developmental dysplasia of the hip between the Mako robotic arm-assisted system and computed tomography-based navigation. Int Orthop.

[14] Domb BG, Chen JW, Kyin C, Bheem R, Karom J, Shapira J (2021). Primary robotic-arm assisted total hip arthroplasty: an analysis of 501 hips with 44-month follow-up. Orthopedics.

[15] Dorr LD, Malik A, Dastane M, Wan Z (2009). Combined anteversion technique for total hip arthroplasty. Clin Orthop Relat Res.

[16] Lewinnek GE, Lewis JL, Tarr R, Compere CL, Zimmerman JR (1978). Dislocations after total hip-replacement arthroplasties. J Bone Jt Surg Am.

[17] Abdel MP, von Roth P, Jennings MT, Hanssen AD, Pagnano MW (2016). What safe zone? The vast majority of dislocated THAs are within the Lewinnek safe zone for acetabular component position. Clin Orthop Relat Res.

[18] Amuwa C, Dorr LD (2008). The combined anteversion technique for acetabular component anteversion. J Arthroplast.

[19] O'Connor PB, Thompson MT, Esposito CI, Poli N, McGree J, Donnelly T (2021). The impact of functional combined anteversion on hip range of motion: a new optimal zone to reduce risk of impingement in total hip arthroplasty. Bone Jt Open.

[20] Widmer K-H (2020). The impingement-free, prosthesis-specific, and anatomy-adjusted combined target zone for component positioning in THA depends on design and implantation parameters of both components. Clin Orthop Relat Res.

[21] Imai H, Miyawaki J, Kamada T, Takeba J, Mashima N, Miura H (2016). Preoperative planning and postoperative evaluation of total hip arthroplasty that takes combined anteversion. Eur J Orthop Surg Traumatol.

[22] Widmer KH, Zurfluh B (2004). Compliant positioning of total hip components for optimal range of motion. J Orthop Res.

[23] Hsu J, de la Fuente M, Radermacher K (2019). Calculation of impingement-free combined cup and stem alignments based on the patient-specific pelvic tilt. J Biomech.

[24] Kutzner KP, Freitag T, Donner S, Kovacevic MP, Bieger R (2017). Outcome of extensive varus and valgus stem alignment in short-stem THA: clinical and radiological analysis using EBRA-FCA. Arch Orthop Trauma Surg.

[25] Leiss F, Götz JS, Meyer M, Maderbacher G, Reinhard J, Parik L (2022). Differences in femoral component subsidence rate after THA using an uncemented collarless femoral stem: full weight-bearing with an enhanced recovery rehabilitation versus partial weight-bearing. Arch Orthop Trauma Surg.

[26] Springer BD, Fehring TK, Griffin WL, Odum SM, Masonis JL (2009). Why revision total hip arthroplasty fails. Clin Orthop Relat Res.

[27] Ulrich SD, Seyler TM, Bennett D, Delanois RE, Saleh KJ, Thongtrangan I (2008). Total hip arthroplasties: what are the reasons for revision?. Int Orthop.

[28] Cruz-Pardos A, Garcia-Cimbrelo E, Cordero-Ampuero J (2005). Porous-coated anatomic uncemented total hip arthroplasty. A 10–17-year follow-up. Hip Int.

[29] Tezuka T, Inaba Y, Kobayashi N, Sato M, Mitsugi N, Saito T (2014). Long-term results of porous-coated anatomic total hip arthroplasty for patients with osteoarthritis of the hip. J Arthroplast.

[30] Hwang K-T, Kim Y-H, Kim Y-S, Choi I-Y (2012). Total hip arthroplasty using cementless grit-blasted femoral component: a minimum 10-year follow-up study. J Arthroplast.

[31] Streit MR, Innmann MM, Merle C, Bruckner T, Aldinger PR, Gotterbarm T (2013). Long-term (20–25-year) results of an uncemented tapered titanium femoral component and factors affecting survivorship. Clin Orthop Relat Res.

[32] Schwarz JS, Lygrisse KA, Roof MA, Long WJ, Schwarzkopf RM, Hepinstall MS (2021). Early, mid-term, and late-term aseptic femoral revisions after THA: comparing causes, complications, and resource utilization. J Arthroplast.

[33] Alpaugh K, Chiu Y-F, Zlotnicki JP, Bendich I, Valle AGD, Bostrom MPG (2022). Femoral component undersizing and alignment are risk factors for early periprosthetic femur fracture. J Arthroplast.

[34] Bozic KJ, Kurtz SM, Lau E, Ong K, Vail TP, Berry DJ (2009). The epidemiology of revision total hip arthroplasty in the United States. J Bone Jt Surg Am.

[35] Brown JM, Mistry JB, Cherian JJ, Elmallah RK, Chughtai M, Harwin SF (2016). Femoral component revision of total hip arthroplasty. Orthopedics.

